# Pre-clinical activity of targeting the PI3K/Akt/mTOR pathway in Burkitt lymphoma

**DOI:** 10.18632/oncotarget.25072

**Published:** 2018-04-24

**Authors:** Maria Bhatti, Thomas Ippolito, Cory Mavis, Juan Gu, Mitchell S. Cairo, Megan S. Lim, Francisco Hernandez-Ilizaliturri, Matthew J. Barth

**Affiliations:** ^1^ Department of Pediatric Hematology/Oncology, University at Buffalo, Buffalo, NY, USA; ^2^ Department of Pediatrics, Roswell Park Cancer Institute, Buffalo, NY, USA; ^3^ Department of Medicine, Roswell Park Cancer Institute, Buffalo, NY, USA; ^4^ Department of Pediatrics, Medicine, Pathology, Microbiology and Immunology, Cell Biology and Anatomy, New York Medical College, Valhalla, NY, USA; ^5^ Department of Pathology and Laboratory Medicine, University of Pennsylvania Perelman School of Medicine, Philadelphia, PA, USA

**Keywords:** Burkitt, PI3K, AKT, idelalisib

## Abstract

Though outcomes for pediatric Burkitt lymphoma (BL) have improved significantly in recent decades with intensive multi-agent chemotherapy and the addition of rituximab, chemotherapy resistance remains a significant impediment to cure following relapse. Activation of the PI3K/AKT pathway has been implicated in Burkitt lymphomagenesis and increased PI3K/AKT activation has been associated with worse outcomes in adults with aggressive B-cell non-Hodgkin lymphoma (B-NHL). Inhibitors of the PI3K/AKT pathway have been approved for the treatment of refractory indolent B-NHL and continue to be investigated for treatment of aggressive B-NHLs. We investigated the activation of the PI3K/AKT pathway in a cell line model of resistant BL and the ability to target this pathway with small molecule inhibitors in BL cell lines. We found that cell lines resistant to rituximab and chemotherapy exhibited increased activation of PI3K/AKT and that inhibition of AKT or PI3K results in *in vitro* anti-lymphoma activity. To investigate the role of PI3K/AKT activation on the efficacy of cytotoxic chemotherapy, we exposed cells to inhibitors in combination with chemotherapy and noted a synergistic increase in response to chemotherapy. Overall these findings highlight the role of PI3K/AKT in chemotherapy resistance in BL cells and may represent a tractable therapeutic target.

## INTRODUCTION

Burkitt lymphoma (BL) is an aggressive form of B-cell non-Hodgkin lymphoma (NHL) and is the most common type of NHL in children. With intense, multi-agent chemotherapy regimens, survival rates for childhood BL have improved significantly in recent decades. Currently, greater than 90% of children diagnosed with BL are cured of their disease [1, 2]. While highly curable, the therapy is quite toxic with high rates of acute toxicity including mucositis and infections. Approximately 2% of children with BL die from toxic complications of therapy [3–5]. In the small percentage of children who develop relapsed or refractory disease, the prognosis is much grimmer with long term survival achieved in only 20–30% of children following further intensified therapy, generally including either autologous or allogeneic hematopoietic stem cell transplant, necessitating the development of novel therapeutic approaches in an attempt to both decrease toxicity in up-front therapy and prolong survival in relapsed/refractory disease [6–9].

Recent publications have reported on genomic alterations observed in BL which contribute to lymphomagenesis. Several recurrent abnormalities have been described by our group and others [10–12]. Schmitz *et al* identified genomic abnormalities in sporadic BL cases and cell lines [13]. Compared to tumor cells from germinal center B-cell (GCB) derived diffuse large B-cell lymphoma (DLBCL), BL tumors harbor recurrent mutations that were distinct from those seen in GCB DLBCL. Along with the expected mutation of the C-MYC proto-oncogene, additional recurrent mutations were observed in in the gene encoding TCF3 and that of its negative regulator ID3 with up to 70% of tumors bearing mutations in one or both of the genes suggesting TCF3 may play a vital role in BL lymphomagenesis. This was further supported by the lethal effects of TCF3 knockdown or ID3 wildtype overexpression in BL cell lines. TCF3 was noted to upregulate components of the B-cell receptor (BCR) pathway leading to activation of the phosphatidylinositol-3-kinase (PI3K) pathway through “tonic” non-NF-kB dependent BCR signaling, rather than the NF-kB dependent chronic active BCR signaling seen in activated B-cell like (ABC) DLBCL, potentially through its effects on the phosphatase SHP-1 which inhibits BCR signaling. Additional data supporting the relevance of the PI3K pathway to BL lymphomagenesis was reported in a recently developed transgenic mouse model and in a proteomic analysis reported by our group [14, 15]. In this model, concurrent activation of both c-Myc and PI3K was noted to lead to lymphoid tumors that morphologically and genetically appear BL-like suggesting the coordination of overexpression of Myc and activation of PI3K may contribute to development of BL.

Overexpression of Myc may further contribute to the activation of PI3K through the Myc dependent induction of microRNAs (miRs) associated with PI3K activation through their inhibitory effect on PTEN, in particular the miR17-92 cluster [16, 17]. Increased expression of Myc-induced miRs has been linked to increased relapse risk in childhood BL. A genome wide copy number analysis of childhood BL samples identified a recurrent gain in the region of 13q31, which incorporates the MIR17HG locus [18]. These samples had higher expression of miR-17 and tended toward early relapse. These findings were further validated by a second report associating increased expression of miR-17 with shorter overall survival (OS) [19].

With the apparent importance of c-Myc and PI3K coordination in BL lymphomagenesis, we investigated the activity of inhibitors of the PI3K/Akt/mTOR pathway in BL cell lines. Numerous inhibitors of this pathway are in clinical development including both narrowly and broadly focused inhibitors in addition to dual inhibitors of both PI3K and mTOR. The more targeted inhibitor of the delta isoform of PI3K, idelalisib, has already gained regulatory approval for the treatment of relapsed chronic lymphocytic leukemia (CLL), small lymphocytic lymphoma (SLL) and follicular lymphoma (FL). In our current contribution, inhibition of the PI3K/Akt/mTOR pathway was investigated in a panel of BL cell lines including cell lines that exhibit a high degree of resistance to both chemotherapy and anti-CD20 immunotherapy.

## RESULTS

**Figure 1:**
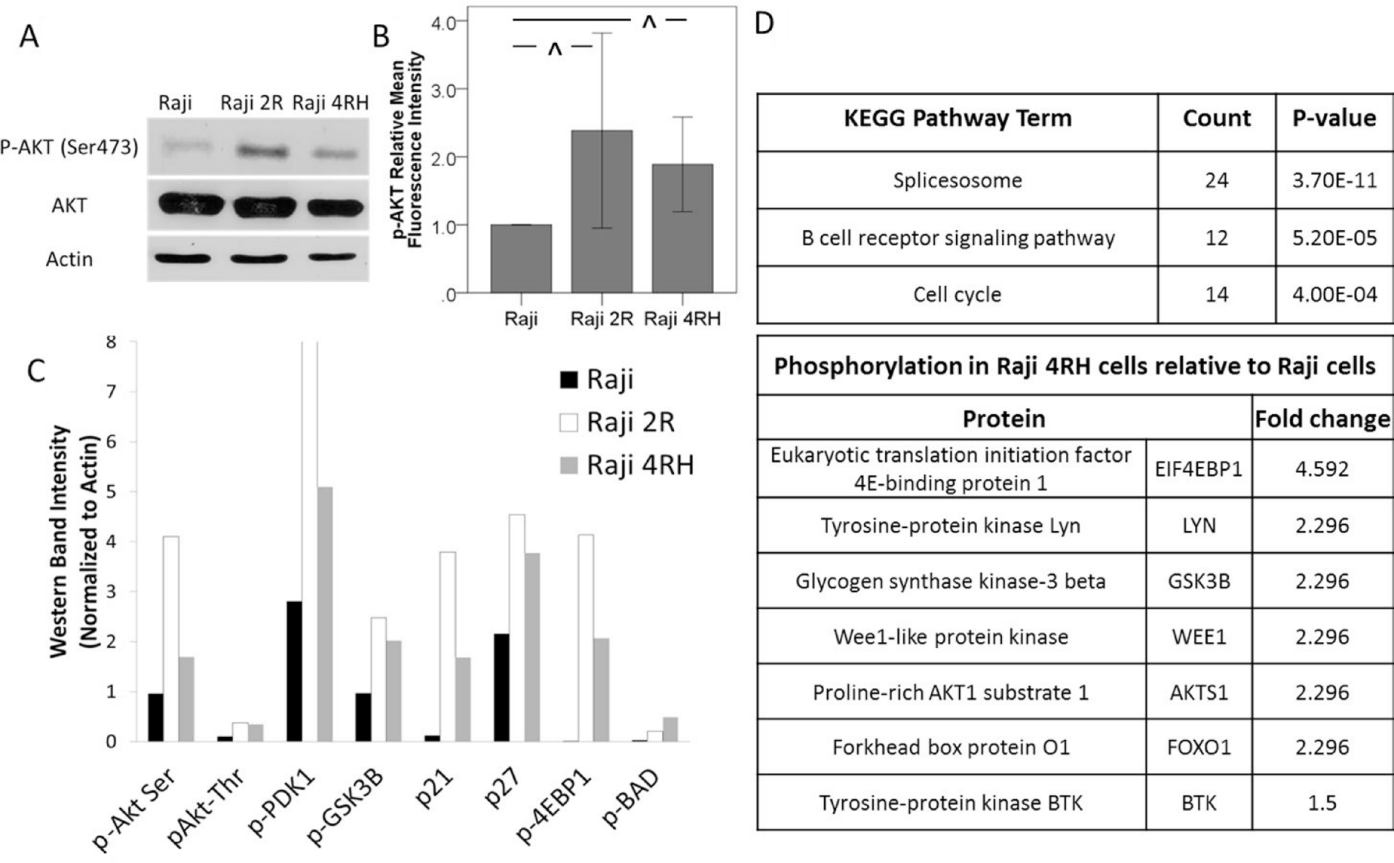
Chemotherapy resistant BL cell lines exhibit an increase in Akt activation Raji 2R and Raji 4RH cell lines derived from the BL Raji cell line demonstrate an increase in Akt phosphorylation by (**A**) Western blot and (**B**) phosphoflowcytometry indicating increased activation of the PI3K/Akt pathway. (**C**) Proteins up and downstream from AKT in the B-cell receptor and PI3K/AKT pathways are differentially phosphorylated in Raji 2R and Raji 4RH cells compared to parental Raji cells on Western blotting. (**D**) Phosphoproteomic analysis identified the B cell receptor signaling pathway as one of the top differentially phosphorylated pathways in Raji 4RH cells with several targets of AKT exhibiting increased phosphorylation. Error bars represent 95% confidence intervals. (^^^*P*-value < 0.03).

With reported evidence of increased Akt activation having a potential impact on survival in B-cell NHL [20–23], we initially characterized the Akt activation in our sensitive and resistant Raji cell lines. On Western blot analysis of p-Akt expression, rituximab-chemotherapy sensitive Raji cells exhibited lower p-Akt expression when compared to the rituximab-chemotherapy resistant Raji 2R and Raji 4RH cell lines (Figure 1A). Similar findings were observed using phospho-flow cytometry, where an approximately 2 fold increase in p-Akt was observed in the resistant Raji cell lines (Figure 1B and Supplementary Figure 1). In order to further evaluate the activation of Akt in these cell lines, the phosphorylation status of downstream targets of Akt was also determined using Western blotting (Supplementary Figure 2). In the resistant cell lines, multiple targets of Akt demonstrated a pattern of increased phosphorylation in the resistant cell lines (Figure 1C). Additionally, in a previously reported phosphoproteomic analysis of parental Raji cells compared to rituximab-chemotherapy resistant Raji 4RH cells, the B-cell receptor pathway was one of the top pathways with significant differential phosphorylation including differential phosphorylation of several proteins both up and downstream of PI3K and Akt associated with PI3K/Akt pathway activation (Figure 1D). These findings indicated a possible role of increased activation of the PI3K/Akt/mTOR pathway in the chemotherapy resistance observed in our resistant BL cells suggesting that targeting this pathway may be therapeutically relevant in BL.

**Figure 2:**
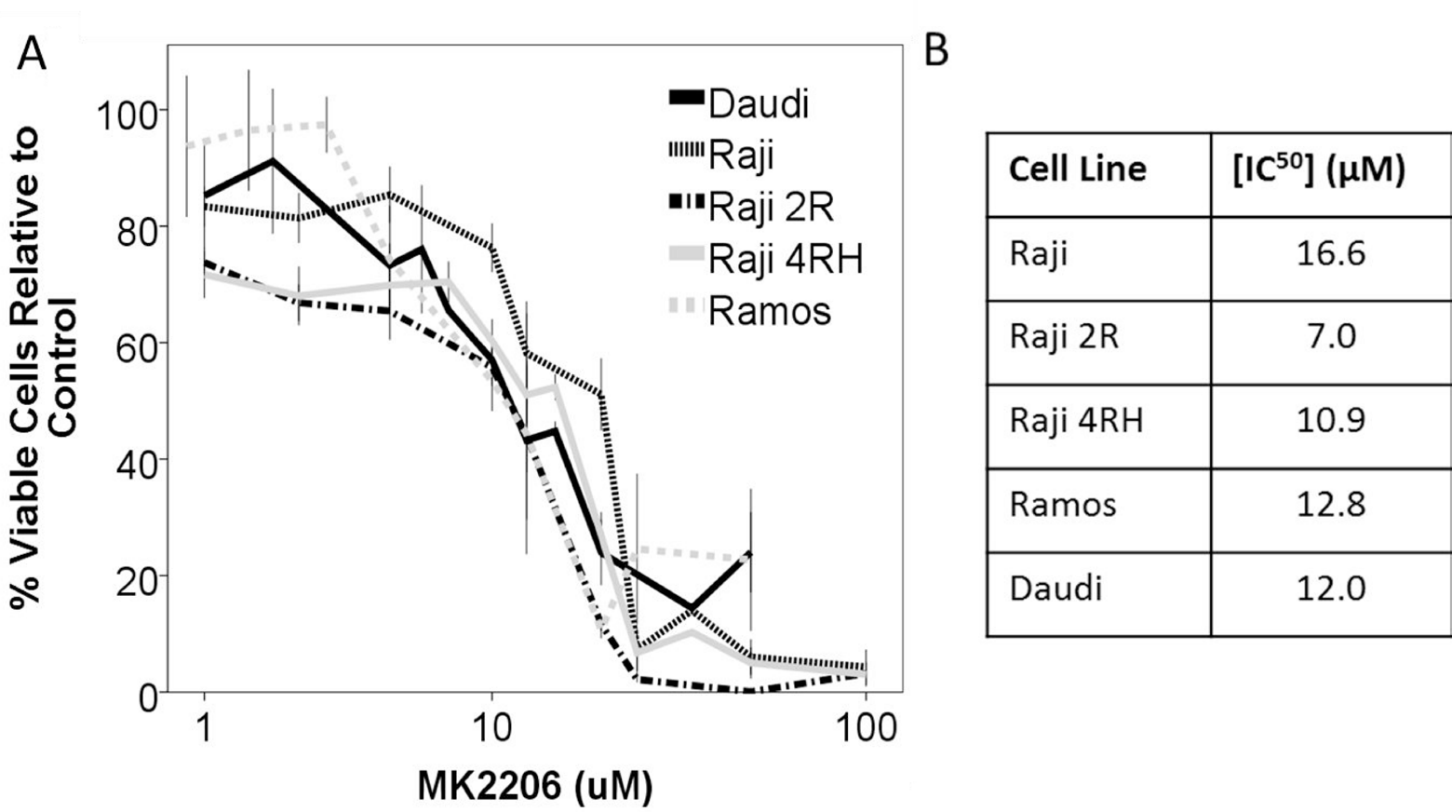
Inhibition of Akt by the inhibitor MK-2206 leads to a decrease in cellular viability in a panel of BL cell lines (**A**) The BL cell lines Raji, Raji 2R, Raji 4RH, Ramos and Daudi were exposed to escalating concentrations of MK2206 for 48 hours and exhibited a dose dependent decrease in viable cells. (**B**) IC50 concentrations at 48 hours. Error bars represent 95% confidence intervals.

**Figure 3:**
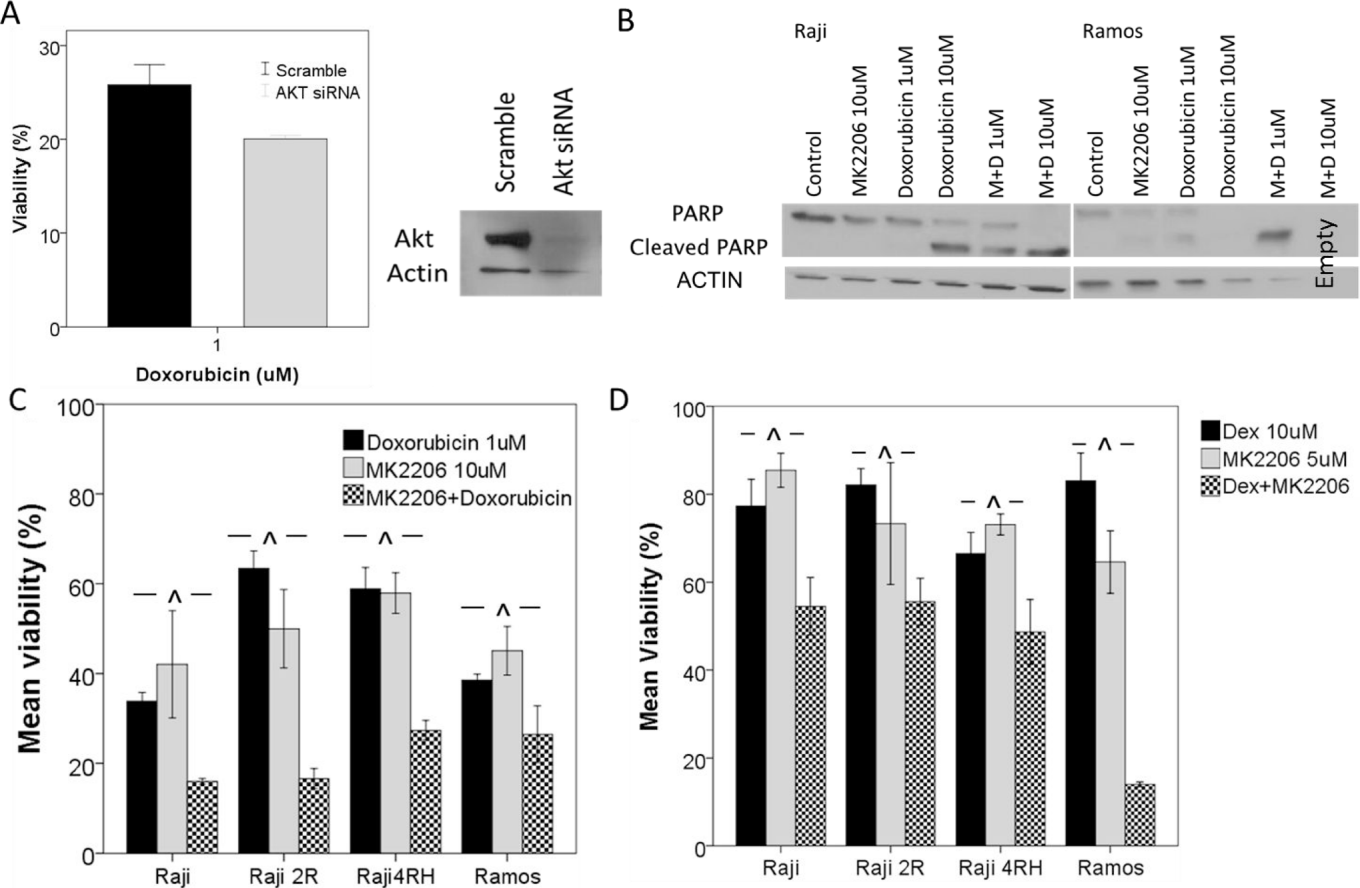
Knockdown of AKT or inhibition of AKT by MK2206 leads to an increase in response to cytotoxic chemotherapy (**A**) Akt knockdown by siRNA led to a decrease in Raji cell viability following 48 hour exposure to doxorubicin. (**B**) BL cell lines were exposed to MK2206 alone or in combination with doxorubicin for 48 hours and apoptosis was measured by Western blot for cleaved PARP. Raji and Ramos cells exhibited little apoptosis following exposure to MK2206 alone however a synergistic increase in PARP cleavage was noted with the combination treatment. *In vitro* exposure to MK2206 and (**C**) doxorubicin or (**D**) dexamethasone leads to a synergistic decrease in cell viability in BL cell lines. Error bars represent 95% confidence intervals. (^^^*p* < 0.05. M = MK2206, D = Doxorubicin, Dex = Dexamethasone).

With Akt being central to activity of the PI3K/Akt/mTOR pathway, we initially investigated the effect of direct Akt inhibition with the pan-Akt inhibitor MK-2206. Burkitt cell lines were exposed to MK-2206 over a range of concentrations and durations. Direct inhibition of Akt by MK-2206 resulted in a decrease in cellular proliferation exhibited in AlamarBlue assays of cell viability compared to control (Figure 2). While a decrease in cell proliferation was observed, determination of apoptosis induction by western blot for cleaved PARP indicated that Akt inhibition by MK-2206 was not leading to significant apoptosis induction as a single agent in any of the Raji cell lines; though a modest increase in PARP cleavage was noted in Ramos cells indicating that this was likely a primarily anti-proliferative effect (Figure 3A).

In order to investigate the hypothesis that PI3K/AKT/mTOR pathway activation contributes to chemotherapy resistance, we investigated targeting AKT either via siRNA knockdown or with MK-2206 in combination with chemotherapeutic agents (Figure 3). Following AKT knockdown, cells were resuspended in fresh medium and cell proliferation and viability were determined after 72 hours using Trypan blue. A decrease in cell proliferation and viability was noted in knockdown cells as compared to cells transfected with a control scramble siRNA. This was particularly noted in the Raji 4RH cells with a decreased number of cells (scramble vs siRNA: 0.41 × 10^6^ vs 0.12 × 10^6^ cells/mL) and viability (82% vs 62%). Following demonstration of Akt knockdown by Western blot, an Alamar Blue assay was performed after 48 h of incubation with doxorubicin. In Raji Akt KD cells, a modest increase in response to doxorubicin was noted with a decrease in viability of KD cells compared to controls (27% vs 19%, *p* < 0.03) (Figure 3A). When BL cell lines were exposed to both MK-2206 and chemotherapy, significant increases in chemotherapy activity were noted. In particular, the combination of MK-2206 with doxorubicin (CI values 0.4–0.7) (Figure 3C) or dexamethasone (CI values 0.3–0.8) (Figure 3D) resulted in significant synergistic reductions in viable cells in AlamarBlue assays when compared to either single agent exposure after normalization to vehicle treated control, as determined using the Chou-Talalay method. Additionally, while single agent MK2206 exposure did not result in significant induction of apoptosis as measured by western blot for PARP cleavage, a synergistic increase in cleaved PARP was noted in combinations of MK-2206 and doxorubicin in both Raji and Ramos cell lines (Figure 3B). In the resistant Raji 4RH cells, however, there was still no significant indication of apoptosis induction. This is likely related to the highly impaired apoptotic machinery in these resistant cell lines, also previously described.

**Figure 4:**
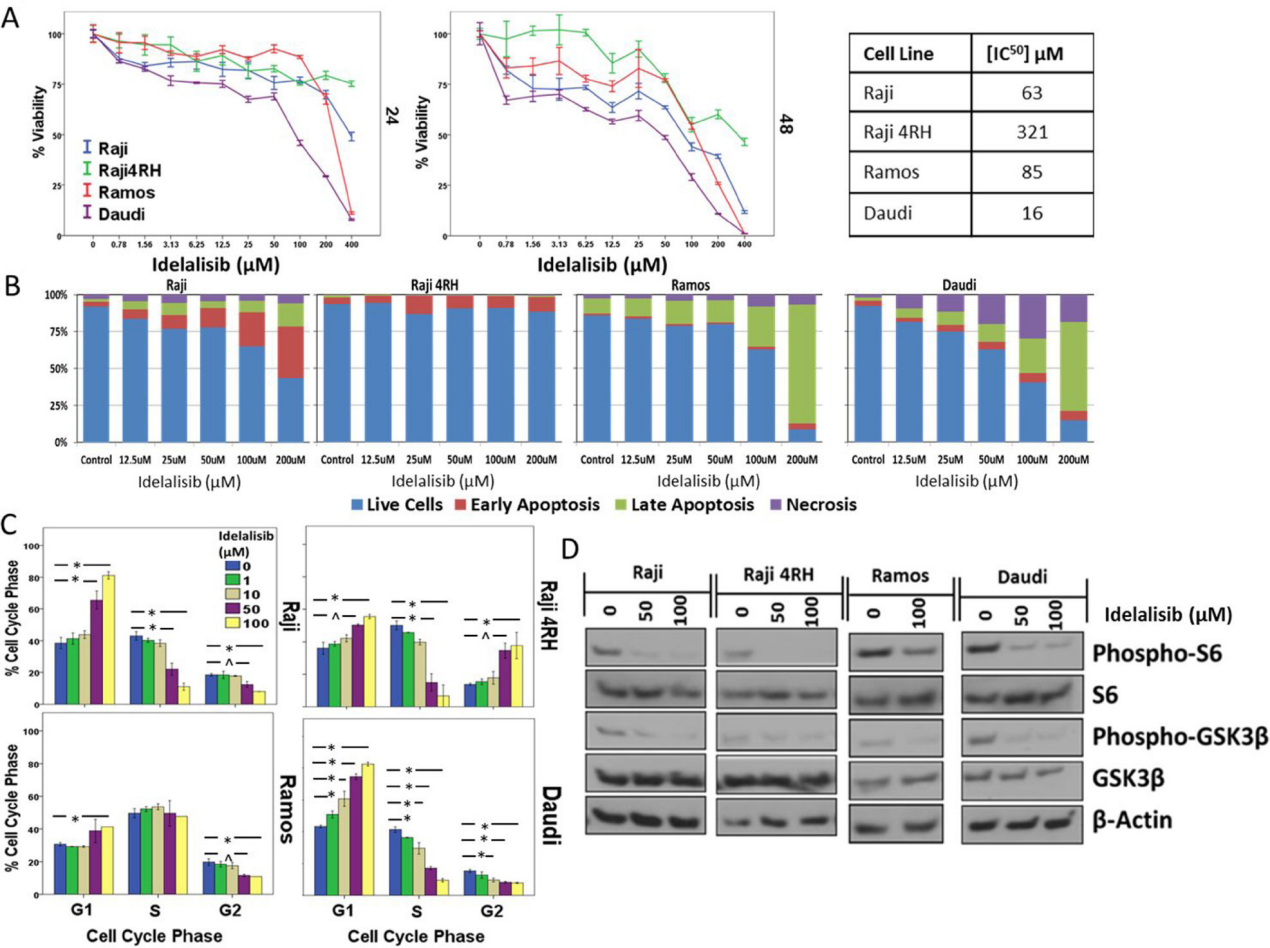
Inhibition of PI3K-delta isoform by idelalisib leads to decreased proliferation and induction of apoptosis in BL cell lines (**A**) *In vitro* exposure to idelalisib for 24 or 48 hours leads to a dose- and time-dependent decrease in BL cell viability and (**B**) induction of apoptosis after 48 hours of exposure. (**C**) Decreased proliferation is associated with an arrest in G1 or G2/M phase of the cell cycle after a 48 hour exposure to idelalisib. (**D**) Idelalisib exposure leads to decreased phosphorylation of downstream targets. Error bars represent 95% confidence intervals. (^*^p < 0.01, ^^^*p* < 0.05).

**Figure 5:**
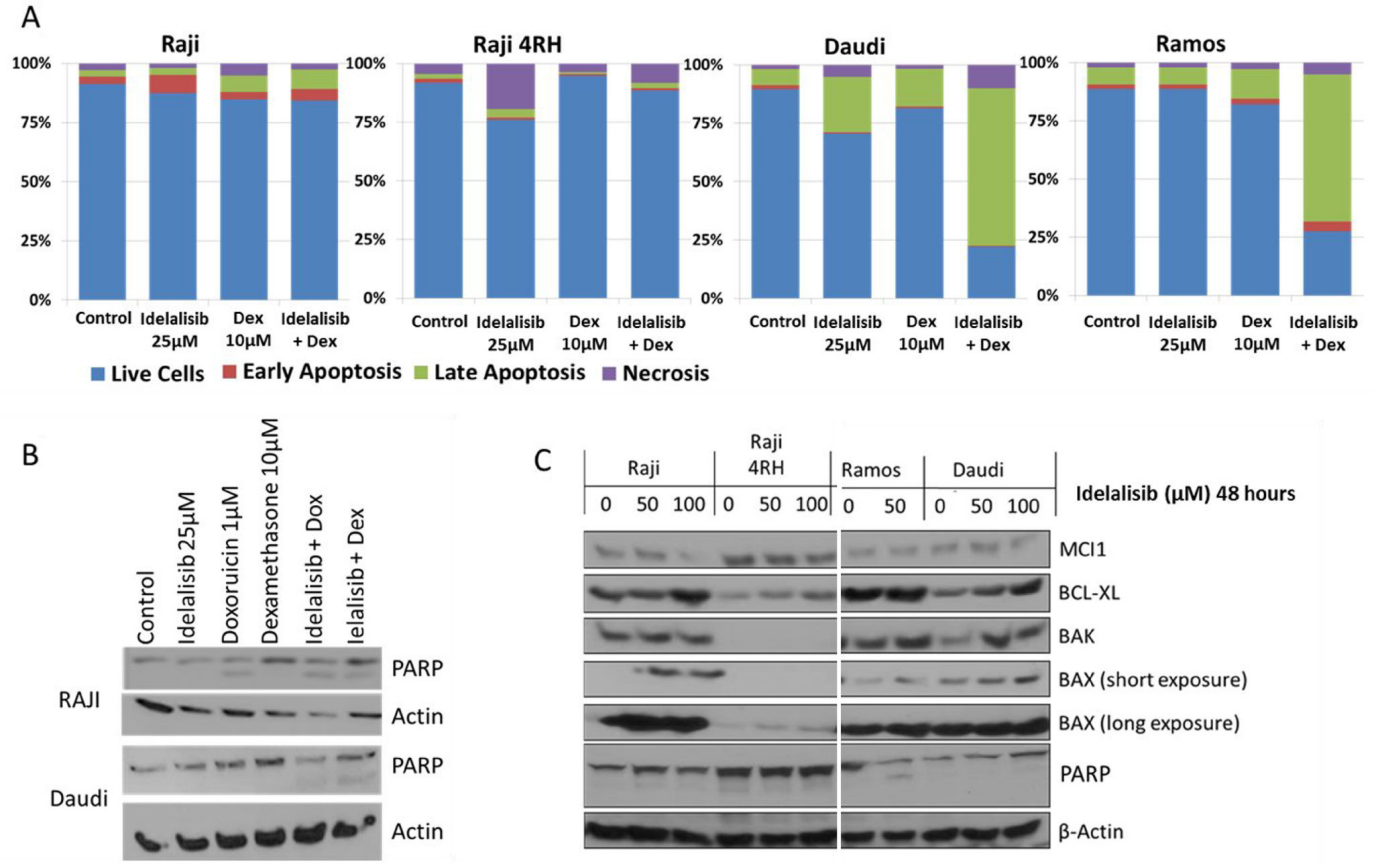
Exposure to idelalisib increases induction of apoptosis in response to cytotoxic chemotherapy (**A**) BL cells exposed to idelalisib and dexamethasone for 48 hours exhibit synergistic induction of apoptosis as measured by flow cytometry for Annexin V and propidium iodide staining in BL Ramos and Daudi cells, but no apoptosis induction in Raji or Raji 4RH cells *in vitro*. (**B**) PI3K inhibition by idelalisib leads to synergistic induction of PARP cleavage when combined with dexamethasone in Raji cells and when combined with dexamethasone or doxorubicin in Daudi cells. (**C**) Idelalisib exposure leads to altered expression of Bcl-2 family regulators of apoptosis. (Dex = Dexamethasone, Dox = Doxorubicin).

To further investigate the role of PI3K/Akt/mTOR pathway inhibition in BL, additional experiments were performed with the PI3K-delta isoform specific inhibitor idelalisib, which is already FDA approved for relapsed/refractory CLL/SLL and FL. Similar to MK-2206, a decrease in BL cell viability was noted with increasing concentrations of idelalisib while Idelalisib exposure also resulted in dose-dependent cell cycle arrest leading to a significant accumulation of cells primarily in G1, though with significant G2/M arrest also noted in resistant Raji 4RH cells (Figure 4C and Supplementary Figure 3). The concentrations required to achieve activity in the BL cell lines tested was well into the μM range with IC^50^ concentrations ranging from 50–300 μM after a 48 h exposure (Figure 4A). At concentrations at or exceeding the IC^50^ concentration, idelalisib did result in induction of apoptosis in all cell lines, though to a significantly lesser degree in the resistant Raji 4RH cells (Figure 4B and Supplementary Figure 4). Inhibition of PI3K-delta with idelalisib inhibited downstream pathway activation as exhibited by decreases in the phosphorylation of downstream targets including pS6 and pGSK3B by western blotting (Figure 4D). Though idelalisib required high concentrations to demonstrate single agent anti-proliferative activity, lower concentrations of idelalisib when combined with dexamethasone led to a synergistic induction of apoptosis, in particular in the relatively more sensitive Ramos and Daudi cell lines (Figure 5A and Supplementary Figure 5). Western blots also demonstrated a synergistic induction of PARP cleavage in Raji cells treated with idelalisib and dexamethasone (Figure 5B). This correlated with changes in expression of Bcl-2 family proteins including an increase in pro-apoptotic Bax and Bak and a decrease in anti-apoptotic MCL-1 leading to an overall more pro-apoptotic pattern of Bcl-2 family protein expression (Figure 5C).

## DISCUSSION

BL is an aggressive form of B-NHL that is highly curable with intensive multi-agent chemoimmunotherapeutic regimens [24–26]. Our group and others have reported that recurrent genetic abnormalities have been observed in children with BL including the critical importance of the PI3K/Akt/mTOR pathway in Burkitt lymphomagenesis [10, 12–14, 18, 19, 27]. Inhibitors of this pathway continue to be investigated in adult lymphoma with most success noted in more indolent lymphoma sub-types. Activity of idelalisib in adults with DLBCL has been very limited.

In our preclinical testing, we demonstrated that, in our cell line model of resistant BL, the PI3K/AKT/mTOR pathway appears to exhibit increased activation. Targeting either AKT or PI3K with pharmacological inhibitors led to primarily an anti-proliferative effect in a panel of Burkitt cell lines with the majority of activity noted at concentrations in the high μM range. In general these concentrations exceed the peak plasma concentrations noted in phase 1 pharmacokinetic studies of these agents in humans [28, 29]. This limited *in vitro* activity at biologically relevant concentrations may relate to the lack of activity noted in single agent trials of idelalisib in aggressive B-cell NHLs.

However, at concentrations below those leading to single agent activity, there was noted to be an increase in apoptosis induction in response to chemotherapy exposure in combination with inhibition of AKT or PI3K. In a report from the Bonavida laboratory, the PI3K/Akt pathway was previously noted to be involved in chemosensitization observed following exposure of BL cell lines to the anti-CD20 monoclonal antibody rituximab [30]. While this previous report implicated a decrease in the anti-apoptotic Bcl-xL protein, we did not observe such a decrease following PI3K inhibition with idelalisib, though we did note changes in expression of other Bcl-2 family proteins including the pro-apoptotic Bax and Bak proteins likely leading to a similar alteration in apoptotic potential following PI3K inhibitor exposure [31]. The possible contribution of increased AKT activation to resistance to rituximab in DLBCL has also been suggested [32]. The interaction of BCL-2 family regulators of apoptosis and activity of the PI3K/AKT/mTOR pathway has been further highlighted by the synergistic activity of BH3 mimetic agents in combination with inhibition of the PI3K/AKT/mTOR pathway with findings of altered MCL-1 and BAX expression following inhibition of PI3K/AKT/mTOR, similar to our results reported here, associated with increased activity of the BCL-2 inhibitor ABT-199 [31, 33, 34]. Additionally, the PI3K/Akt/mTOR pathway has been reported to play a central role in the activity of a variety of targeted therapeutic agents under pre-clinical investigation in aggressive B-cell NHL, in many cases through alterations in BCL-2 family protein expression [35–38].

While single agent inhibition of PI3K has demonstrated promise in targeting B-NHL, the investigation of agents targeting the PI3K/AKT/mTOR pathway with enhanced activity has also led to the development of numerous inhibitors more broadly targeting PI3K (compared to the delta isoform specific idelalisib) or targeting both up- and down-stream targets in the pathway, such as through dual targeting inhibitors of PI3K and mTOR. Such agents have exhibited pre-clinical and clinical activity expanding the repertoire of agents targeting this pathway [39–43].

Though PI3K inhibitors have led to limited single agent activity in aggressive adult B-cell lymphomas, considering the data suggesting synergistic activity of PI3K/AKT/mTOR inhibition, a more rational application may be in the setting of combination therapy along with cytotoxic chemotherapy or other targeted agents. A potential area of concern in regards to the use of agents targeting this pathway is an apparent high rate of toxicities, including potentially serious gastrointestinal, hepatic and pulmonary toxicities, that have been observed in adult patients being treated with idelalisib or the Bruton’s tyrosine kinase inhibitor ibrutinib, in particular when utilized in combination with other therapeutic agents [44, 45]. This may impair the ability to combine these agents with the types of chemotherapy agents typically used in treating BL. Additionally, despite the dismal prognosis for such patients, the rarity of childhood relapsed/refractory BL along with the sheer number of available agents currently under investigation in adults (including numerous agents targeting the PI3K/Akt/mTOR pathway) complicates the choice of which agents to pursue clinically, making the establishment of biological rationale critical to future clinical investigation of novel therapies in pediatric B-NHL.

In summary, targeting PI3K appears to have biological relevance in BL and *in vitro* targeting of the PI3K/Akt/mTOR pathway in models of BL exhibits promising pre-clinical activity validating further pursuit of novel therapeutic agents targeting this pathway in BL. Though initial clinical trials of idelalisib in adults with DLBCL have been less promising, the activity of narrow versus broad inhibition of PI3K, further upstream inhibition with inhibitors of the B-cell receptor or dual inhibition of both up and downstream targets within the pathway also continue to be investigated and may hold additional promise of clinical efficacy, while observed toxicity continues to be a potential limiting factor to incorporation of these agents to the intense chemotherapy regimens generally used in treating aggressive childhood B-NHLs and needs to be further characterized.

## MATERIALS AND METHODS

### Cell lines and culture

BL cell lines were used for the experiments including Raji, Ramos and Daudi and were purchased from American Type Culture Collection (ATCC, Manassas, VA, USA). Rituximab resistant cell lines Raji 2R and Raji 4RH were created and characterized from parental Raji cells as previously described [46, 47]. All cell lines were maintained in RPMI 1640 with Glutamax-1 (Invitrogen, Karlsruhe, Germany) supplemented with 10% heat-inactivated fetal bovine serum (FBS), HEPES (5 mmol/l), penicillin and streptomycin at 100 IU/ml and sodium pyruvate 1 mmol/l.

### Reagents and antibodies

The AKT inhibitor (MK-2206) and the PI3K-delta inhibitor (idelalisib) were purchased from Selleck Chemicals (Houston, TX, USA). Cisplatin was purchased from American Pharmaceutical Partners (Schaumburg, IL, USA). Doxorubicin was obtained from Bedford Labs (Bedford, OH, USA) and dexamethasone was provided by the Roswell Park Cancer Institute (RPCI) Pharmacy.

Primary mouse anti-human antibodies raised against BAK, BAX, BCL-XL, MCL-1, PARP, AKT, p-AKT, GSK3B, p-GSK3B, S6, p-S6, PDK1, p-PDK1, p27, p21, BAD, p-BAD, 4-EBP1, p-4-EBP1 and actin were purchased from Cell Signaling Technologies (Danvers, MA, USA). Alkaline phosphatase (AP) or horseradish peroxidase (HRP) conjugated anti-mouse secondary antibodies were purchased from Jackson ImmunoResearch (West Grove, PA, USA). Ficoll-Hypaque was purchased from Sigma-Aldrich Inc. (St. Louis, MO, USA). Sodium chromate^51^ (^51^Cr) (Perkin-Elmer Life Inc., Boston MA) was utilized in immunological assays assessing antibody-dependent cellular cytotoxicity (ADCC) and complement mediated cytotoxicity (CMC). Triton X-100, trypan blue and histopaque-1077 were obtained from Sigma-Aldrich Inc. (St Louis, MO). Cell Titer-Glo Luminescent Viability Assay reagent was purchased from Promega (Madison, WI, USA). PrestoBlue cell proliferation assay reagent was purchased from (Invitrogen, Grand Island, NY, USA). AKT siRNA (ON-TARGET plus Smart Pool siRNA) was purchased from Dharmacon (Lafayette, CO, USA).

### Transient siRNA AKT knockdown

A transient AKT siRNA knockdown was performed in Raji and Raji 4RH cells. Cells were cultured in RPMI1640 with 10% HIFBS at 37° C in 5% CO_2_. Cells were passaged 3 days before experimental start and in log rhythmic growth phase. 2 × 10^6^ cells were pelleted, washed and combined with 100uL Amaxa Nucleofector Solution (Lonza, Walkersville MD, USA) and then transferred to an Amaxa cuvette where siRNA was added at a concentration of 300 nM/sample. Samples were then electroporated using Amaxa Nucleofector Devise (Lonza, Walkersville MD, USA) using the M-13 protocol. After electroporation cells were transferred back into media and allowed to recover and presence of siRNA knockdown was detected via western blot.

### Phosphoproteomic analysis

Mass spectrometry-based label-free quantitative phosphoproteomic profiling of the BL cell lines Raji and Raji4RH was performed as previously described [48, 49]. Briefly, six milligrams of protein from each cell line were digested by trypsin and peptides were subjected to phosphopeptide enrichment using metal oxide affinity chromatography (MOAC) and immunoprecipitation. An LTQ Orbitrap XL in-line with a Paradigm MS2 HPLC was employed for acquiring high-resolution MS and MS/MS data that were searched with the Swissprot Human taxonomic protein database.

### Changes in expression of PI3K/Akt target proteins and Bcl-2 family members by Western blot

Following relevant drug exposure to PI3K/Akt inhibitors for 24 hours, cells were lysed with a RIPA buffer containing 2 mM PMSF, 1 μg/ml of leupeptin, 1 μg/ml pepstatin and 1 μg/ml aprotinin. After solubilization at 4° C × 60 minutes, nuclei and debris were pelleted at 10,000 rpm for 30 minutes. Protein was quantified using Opsys MR (Thermo LabSystems Inc., Beverly, MA, USA). Lysates were prepared with equal amount of protein, distilled water and 4× laminar buffer. Lysate was loaded onto a 12% sodium dodecyl sulfate-polyacrylamide gel electrophoresis (SDS-PAGE) gel and transferred onto a nitrocellulose membrane using iBLOT (Invitrogen Technologies, Grand Island, NY, USA). The membrane was blocked for a minimum of 1 hour with 5% milk in PBS and then incubated at 4° C overnight with antibodies directed against proteins of interest. After adding the appropriate AP- or HRP-conjugated secondary antibody, detection was performed using enhanced chemiluminescence visualization system (ECL, plus, Amersham Life Sciences, Arlington Heights, IL, USA). Western blots were performed on three occasions and representative results are depicted.

### *In vitro* effects of PI3K pathway inhibition on the viability of BL cell lines

Cell lines were exposed to escalating doses of MK-2206 (1–50 uM) or idelalisib (0.1–400 μM) or vehicle control (DMSO) for 48 hours. Cells were plated at a cell density of 0.25 × 10^6^ cells/ml in 96 or 384 well plates and at each time period 20 μL of Alamar blue was added to each well and then incubated for 2 additional hours. Cell proliferation was determined as the change in Alamar blue reduction by living cells and measured using a Fluoroskan Ascent LF (Thermo Fisher Scientific, Barrington, IL, USA).

### *In vitro* effects of MK-2206 or idelalisib on the anti-tumor activity of chemotherapy agents

BL cells were placed in 384 well plates (1 × 10^5^ cells/well, cell density of 0.5 × 10^6^ cells/ml) and exposed to idelalisib (1–50 uM) and/or escalating doses of cisplatin (0–60 μM), doxorubicin (0–10 nM), vincristine (0–1 nM) or dexamethasone (1–100 uM). The cells were then incubated at 37° C and 5% CO_2_ for 48 hours and changes in ATP levels as surrogate marker of viability, was measured using the CellTiter Glo luminescence assay. Values for exposures were normalized to vehicle treated control for each cell line tested.

### Cell cycle progression of BL cells exposed to MK-2206 or idelalisib

Propidium iodide (PI) nuclear staining was utilized to determine the cell-cycle fractions (Sigma-Aldrich, St. Louis, MO, USA). Briefly, cells were exposed idelalisib (1, 10, 50 or 100 μM) or DMSO control for 48 hours and subsequently harvested, washed three times in PBS, fixed in 70% ethanol for 30 minutes on ice and then incubated in a PBS/ribonuclease solution for 30 minutes at 37° C (1mL PBS & 10 μM ribonuclease). Data was collected on a FACSCalibur flow cytometer (BD Biosciences; San Jose, CA, USA) and analyzed with Mod Fit LT Version 3.2 software (Verity Software House, Topsham, ME, USA). Results represent the mean value of three independent experiments.

### Determination of apoptosis induction in BL cell lines exposed to MK2206 or idelalisib

Cell lines were exposed to MK2206 or idelalisib for 48 hours in a 6 well plate at 0.5 × 10^6^ cells/ml. Apoptosis was analyzed using Western blotting (as above) using anti-PARP antibody and/or flow cytometry by Annexin V-propidium iodide staining.

### Statistics

Graphic analysis was performed using IBM SPSS 21. Significance was determined using a student’s *T* test. Synergy in combination experiments was determined by the Chou-Talalay method using CalcuSyn software and reported as Combination Index (CI) with values <0.9 indicating synergy and values >1.1 indicating antagonism.

## SUPPLEMENTARY MATERIALS FIGURES


