# Supplementary material for: Pre-clinical activity of targeting the PI3K/Akt/mTOR pathway in Burkitt lymphoma
Source: Oncotarget. 2018 Apr 24;9(31):21820–30. doi: 10.18632/oncotarget.25072 (PMC5955151; doi:10.18632/oncotarget.25072)
Supplement: Supplementary file 1 [file oncotarget-09-21820-s001.pdf]

Pre-clinical activity of targeting the PI3K/Akt/mTOR pathway in Burkitt lymphoma

SUPPLEMENTARY MATERIALS

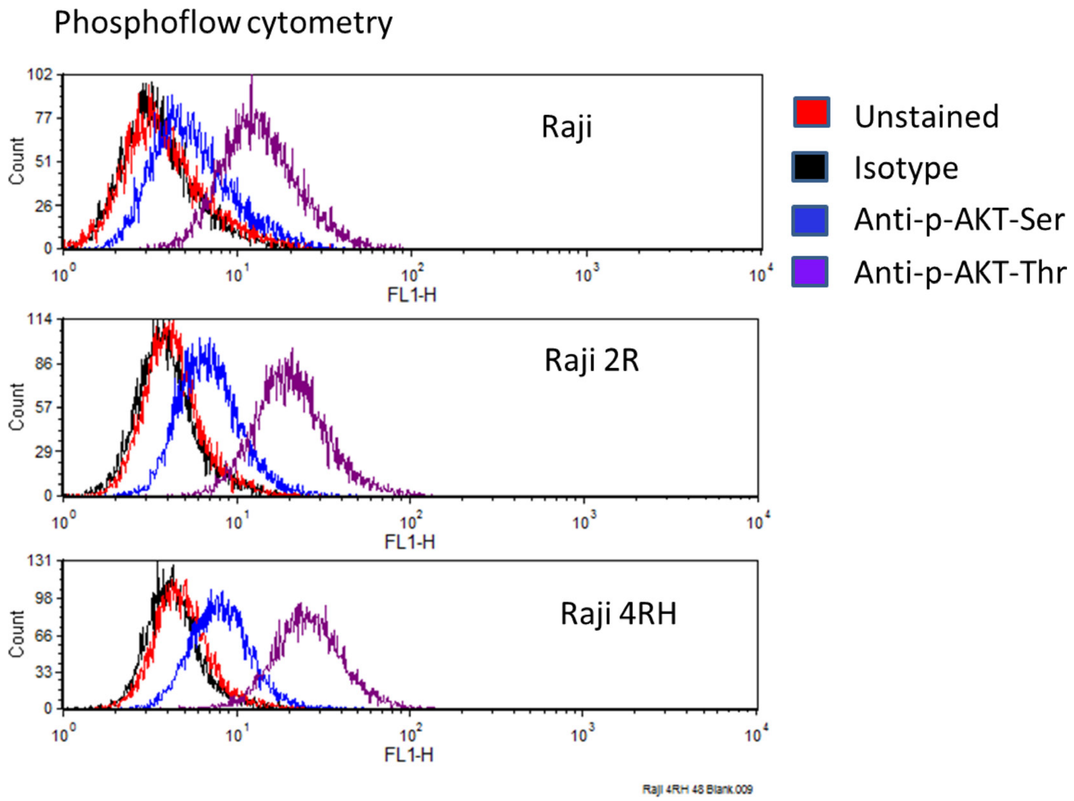

Supplementary Figure 1: Phosphoflow cytpometry for p-AKT in Raji, Raji 2R and Raji 4RH cell lines.

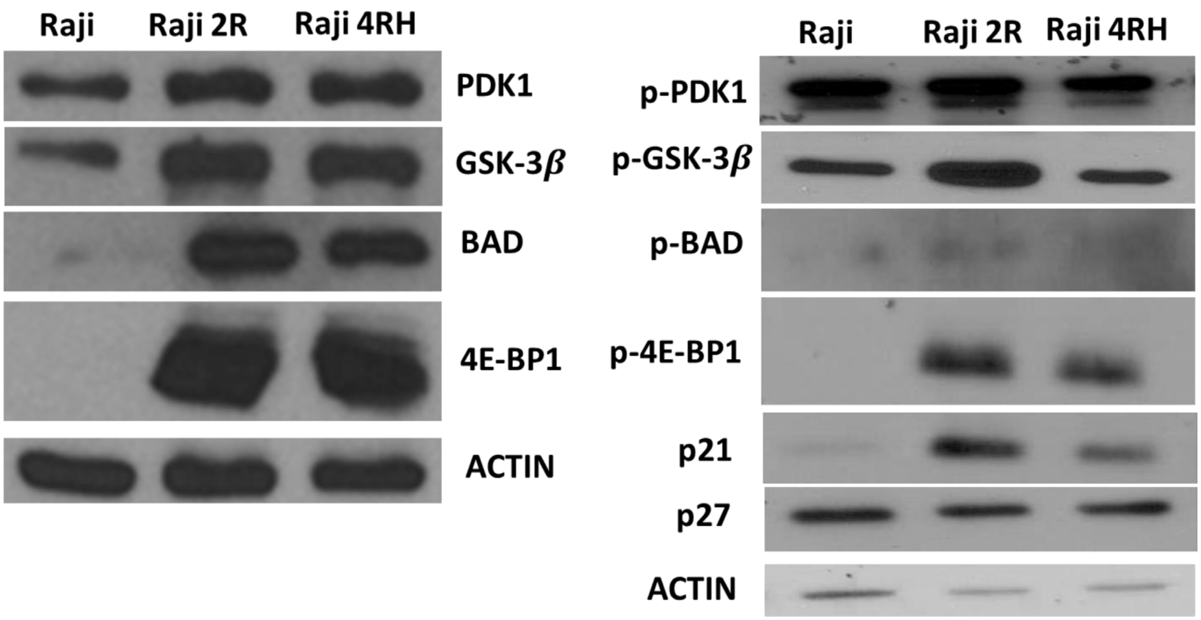

Supplementary Figure 2: Western blots for total and phosphorylated proteins in Raji, Raji 2R and Raji 4RH cells.

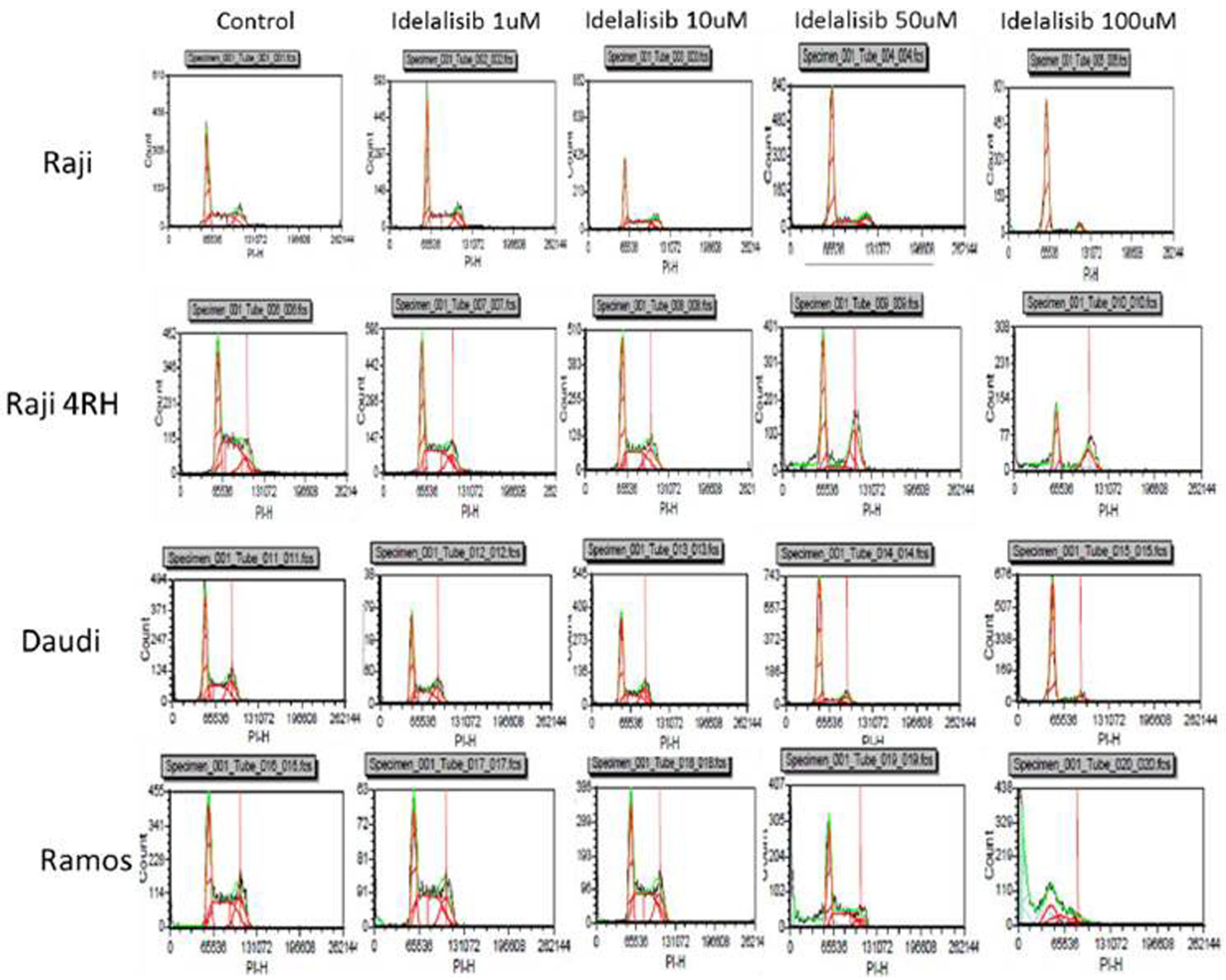

Supplementary Figure 3: FACS plots for cell cycle distribution following exposure to idelalisib in Raji, Raji 4RH, Daudi and Ramos.

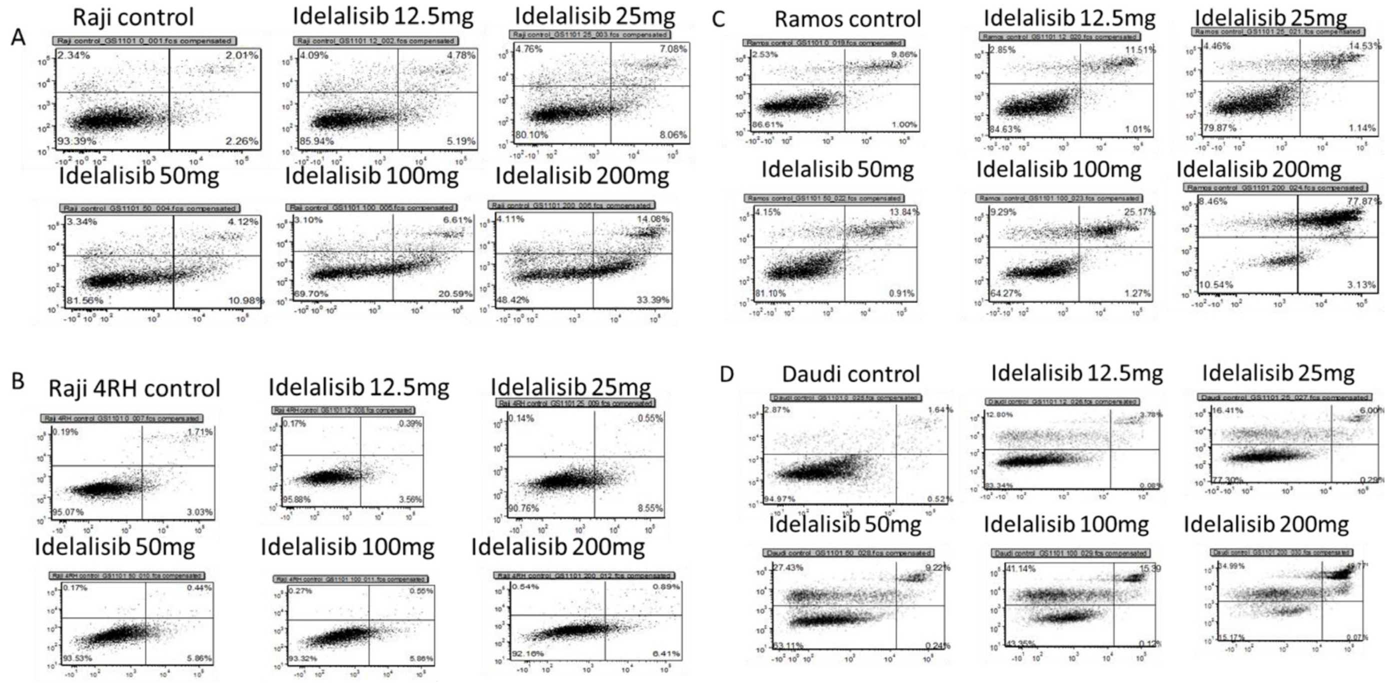

Supplementary Figure 4: FACS plots for apoptosis analysis following exposure to idelalisib in (**A**) Raji, (**B**) Raji 4RH, (**C**) Ramos and (**D**) Daudi.

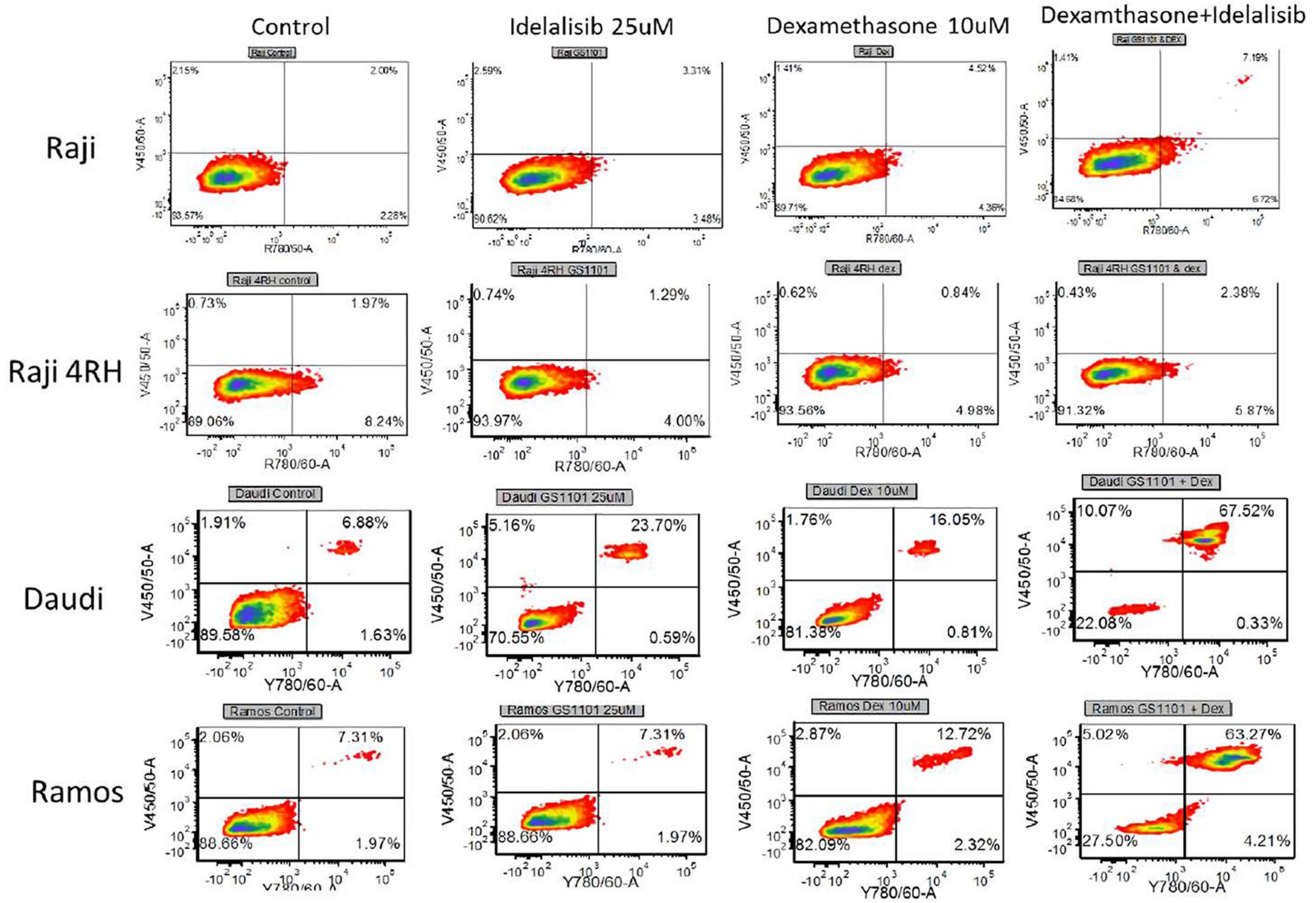

Supplementary Figure 5: **FACS plots for apoptosis analysis following exposure to idelalisib, dexamethasone or the combination of idelalisib and dexamethasone in Raji , Raji 4RH, Daudi and Ramos.**
